# From Serum to Surgery: The Significance of Albumin in Preoperative Risk Stratification—An Analysis of 200,015 Plastic Surgery Patients

**DOI:** 10.1007/s00266-026-05800-8

**Published:** 2026-03-17

**Authors:** Michael Alfertshofer, Samuel Knoedler, P. Niclas Broer, Philipp Moog, Hans-Günther Machens, Konstantin Frank, Leonard Knoedler, Max Heiland, Carsten Rendenbach, Steffen Koerdt, Dennis P. Orgill, Gabriel Hundeshagen, Adriana C. Panayi

**Affiliations:** 1 Department of Plastic and Hand Surgery, Klinikum rechts der Isar, Technical University Munich, Munich, Germany; 2 Department of Oral and Maxillofacial Surgery, Charité Universitätsmedizin Berlin, Berlin, Germany; 3 Division of Plastic Surgery, Brigham and Women’s Hospital, Harvard Medical School, Boston, MA USA; 4 Department of Plastic, Hand and Burn Surgery, Klinikum Bogenhausen, Technical University Munich, Munich, Germany; 5 Department of Plastic, Hand and Reconstructive Surgery, University Hospital Regensburg, Regensburg, Germany; 6 Department of Hand, Plastic and Reconstructive Surgery, Burn Center, BG Trauma Center Ludwigshafen, University of Heidelberg, 67071 Ludwigshafen, Germany

**Keywords:** Albumin, Laboratory values, Risk stratification, Complication management, Routine examination

## Abstract

**Background:**

Plastic and reconstructive surgery (PRS) encompasses a wide range of procedures, and postoperative complications remain a persistent challenge. While preoperative laboratory values (PLVs) are routinely assessed, their predictive value for postoperative outcomes in PRS is unclear. This study evaluates the association between PLVs and postoperative morbidity to enhance risk stratification.

**Methods:**

The ACS-NSQIP database (2008–2022) was analyzed for PRS patients. PLVs included sodium, blood urea nitrogen, creatinine, albumin, bilirubin, SGOT, alkaline phosphatase, white blood cell count, hematocrit, platelets, partial thromboplastin time, international normalized ratio, and prothrombin time (PT). Multivariate logistic regression identified independent predictors of complications, while decision tree analysis established risk thresholds, validated through cross-validation.

**Results:**

A total of 200,015 patients with a mean age of 50.3±14.5 years and a mean BMI of 29.1±6.8 kg/m^2^ were included in this study. Albumin levels emerged as the strongest predictor for the occurrence of any complications. Patients with albumin ≤ 3.3 g/dL had a 36.5% risk for the occurrence of any complication compared to 10.4% in those with higher levels. In the high-risk group, prolonged PT (> 16.2s) further increased risk for the occurrence of any complications. Also, in the lower-risk group (albumin > 3.3 g/dL), prolonged PT (> 13.7 s) were found to significantly influence complication occurrence. Further, multivariate analysis confirmed reduced albumin (OR 0.460) as the strongest independent predictors for any complication occurrence.

**Conclusion:**

Albumin and PT are key predictors of 30-day postoperative complications in PRS. Preoperative optimization of these values can enhance patient safety and surgical outcomes. Findings support integrating targeted preoperative evaluations into clinical practice.

**Level of Evidence III:**

This journal requires that authors assign a level of evidence to each article. For a full description of these Evidence-Based Medicine ratings, please refer to the Table of Contents or the online Instructions to Authors www.springer.com/00266.

**Supplementary Information:**

The online version contains supplementary material available at 10.1007/s00266-026-05800-8.

## Introduction

Plastic and reconstructive surgery (PRS) aims to restore both form and function. Its scope includes a wide range of procedures, from aesthetic enhancements to complex reconstructions following trauma or oncologic resection, rendering it one of the most diverse surgical specialties [1–3]. Despite its transformative potential, as with any surgical discipline, complications remain an inherent aspect of clinical practice. Accurately predicting and ultimately minimizing the risk of such is pivotal for advancing the specialty and ensuring patient safety [4–6].

Efforts to minimize surgical and perioperative medical complications have been a global priority across all surgical specialties. Standardized education, the adoption of evidence-based standard operating procedures (SOPs), and critical analysis of clinical workflows including surgical techniques have become instrumental in improving outcomes [7, 8]. Additionally, the widespread integration of digital tools has revolutionized perioperative care, enabling more sophisticated monitoring and individualized patient management [9, 10].

One cornerstone of preoperative evaluation is the routine assessment of laboratory values. For the preponderance of surgical procedures requiring general anesthesia, a standard set of blood tests—commonly including hematocrit (HCT), coagulation parameters, and, in specific cases, assessments of renal function—is performed [11, 12]. These tests provide a snapshot of a patient’s physiological status and help identify conditions that may increase the risk of adverse outcomes. However, the selection of these laboratory parameters often depends rather on institutional norms or surgeon preference than robust evidence-based guidelines [13].

In the field of PRS, particularly, there is a paucity of large-scale studies assessing the predictive value of routine preoperative laboratory measurements for complications in the peri- and postoperative setting. To date, surgical risk stratification has traditionally focused on factors such as patient demographics [14, 15], comorbidities [16, 17], and procedure complexity [18, 19]. While these determinants undoubtedly play a major role, laboratory values offer an additional, quantifiable method of evaluating risk. Due to their widespread use and their ability to reflect various organ systems and their interactions, routine laboratory tests may hold untapped potential that can readily be leveraged to improved surgical risk stratification. Integrating these data into preoperative planning could enhance patient safety and improve outcomes, yet their specific role in PRS remains underexplored.

To our knowledge, no study has systematically analyzed the relationship between routine preoperative laboratory measurements and the incidence of complications in PRS. This investigation aims to bridge that gap, providing evidence to refine preoperative protocols, improve risk stratification, and ultimately enhance patient care in PRS.

## Material and Methods

### Data Source

All data were sourced from the American College of Surgeons—National Surgical Quality Improvement Program (ACS-NSQIP) database, a leading initiative aimed at enhancing the quality of surgical care by providing hospitals with risk-adjusted, outcomes-based data. Established in the early 2000s, more than 700 hospitals across the U.S. and internationally now participate in the NSQIP. The data, directly captured from patients' medical records by trained clinical reviewers, cover a wide range of metrics, including preoperative demographics, intraoperative variables, and 30-day postoperative outcomes. To ensure the highest standards of data quality, ACS-NSQIP uses standardized definitions, strict data collection protocols, and conducts regular audits of participating hospitals [20]. This process enables hospitals to benchmark their performance and identify areas for improvement, thus ultimately optimizing surgical care delivery. All recordings are de-identified to protect patient privacy and confidentiality. Although all data in this study were anonymized, ethical approval was obtained from Brigham and Women’s Hospital, Boston, MA, USA (protocol number: 2013P001244).

### Patient Selection

At the time of analysis, no data more recent than 2022 were available. Records prior to 2008 were excluded due to differing data format and collection methodology. Fifteen consecutive annual datasets from 2008 to 2022 were filtered to include only cases operated on by the surgical specialty “plastic surgery.” This set was subsequently further refined by excluding cases with inaccurate, incomplete, or conflicting coding, as well as obvious miscoding. All cases were manually reviewed. The ACS-NSQIP database captures only surgical cases of patients aged 18 years or older, hence pediatric patients were excluded by design. Cases with a BMI below 7 kg/m^2^ or greater than or equal to 250 kg/m^2^ were excluded from the analysis, as such values are considered physiologically nonviable.

**Fig. 1:**
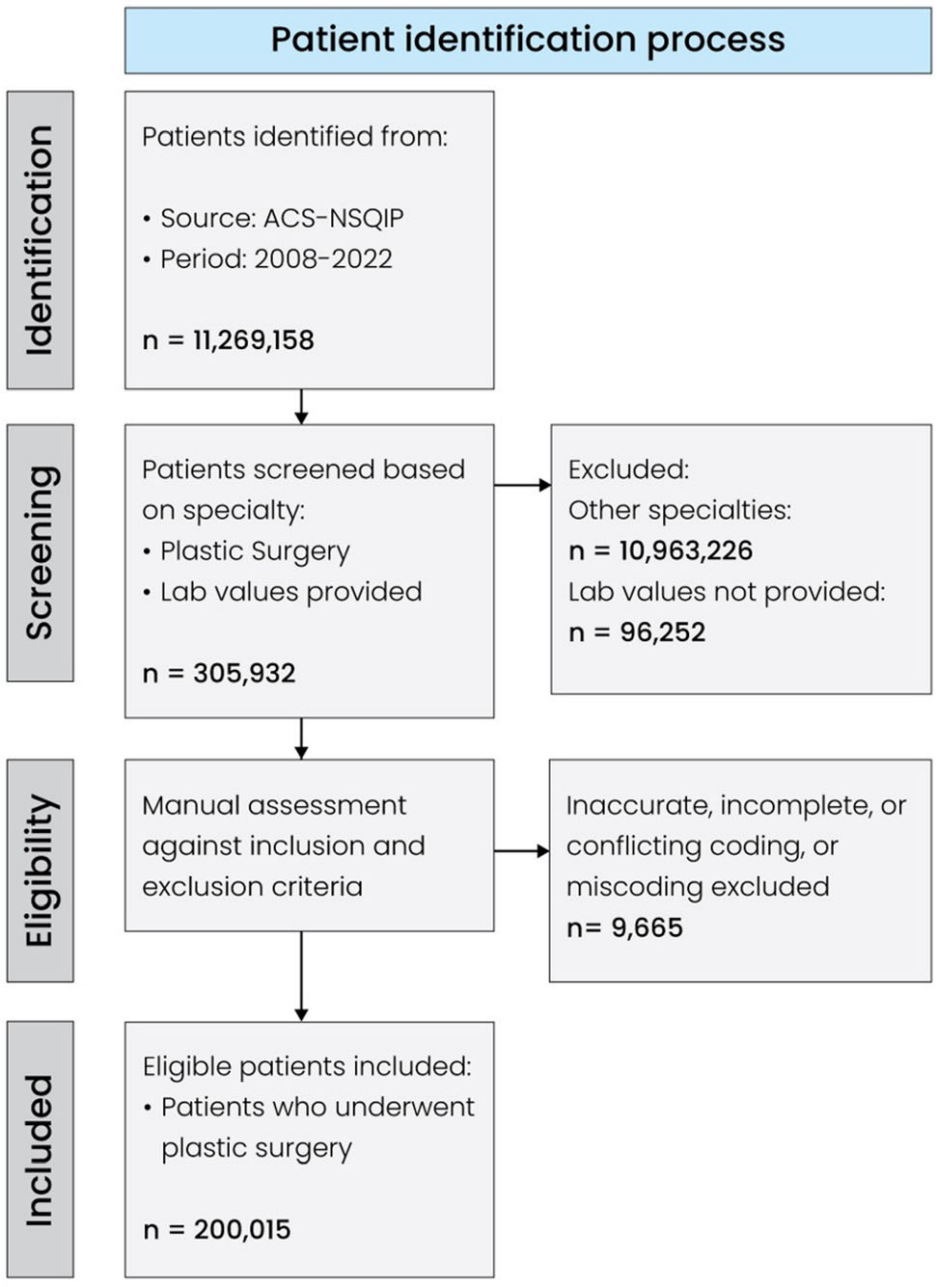
Patient selection process

Therefore, cases with a calculated BMI falling outside these thresholds were deemed likely to be miscoded and were excluded from the analysis. Further, all cases without any reported PLVs were excluded. The final dataset represented adult patients who underwent PRS during a 15-year study period and had at least one PLV reported (Fig. 1).

### Variable Extraction

Pre- and 30-day post-operative parameters were extracted.

(i) We evaluated the following preoperative variables: (a) patient demographics (self-reported gender [male, female, and non-binary], age, self-reported race [American Indian or Alaskan Native, Asian, Native Hawaiian or Pacific Islander, Black or African American, White, and other/unknown], self-reported ethnicity [non-Hispanic and Hispanic], height [in inches], and weight [in pounds]), allowing for calculation of the body mass index (BMI) using the formula [weight (pounds)/height (inches)^2^ × 703], with a BMI ≥30 kg/m^2^ indicating obesity. (b) preoperative laboratory values (serum sodium, blood urea nitrogen [BUN], serum creatinine, serum albumin, total bilirubin, serum glutamic-oxaloacetic transaminase [SGOT], alkaline phosphatase [AP], white blood cell [WBC] count, hematocrit, platelet count, partial thromboplastin time [PTT], international normalized ratio [INR], and prothrombin time [PT].
(ii) As 30-day postoperative outcomes, we collected the length of hospital stay (measured in days between admission and discharge), operating time (in minutes), and discharge destination (home, not-home, or other/unknown). Any complication was defined as the occurrence of either mortality, reoperation, (unplanned) readmission, or any of the following surgical or medical complications. We evaluated all surgical complications systematically reported in the ACS-NSQIP database over the 15-year period, namely superficial and deep incision site infection, organ space infection, wound dehiscence, and excessive bleeding. Likewise, all medical complications captured in the registry, including pneumonia, reintubation, pulmonary embolism, ventilator dependence > 48 hours, progressive renal insufficiency, renal failure, urinary tract infection, stroke/cerebrovascular accident, blood transfusion, cardiac arrest, myocardial infarction, deep vein thrombosis/thrombophlebitis, sepsis, and septic shock, were analyzed for this investigation.

### Statistical Analysis

The raw data available from the ACS-NSQIP were exported to Microsoft Excel files (Version 16, Microsoft Corporation, Redmond, WA, USA) via SPSS for Windows (Version 29, IBM Corporation, Armonk, NY, USA). All records were processed into a standardized format for further analysis, stored in an electronic laboratory notebook (LabArchives, LLC, San Marcos, CA, USA) and analyzed using SPSS Statistics 27 (IBM, Armonk, NY, USA). Differences were considered statistically significant at a probability level of ≤ 0.05 to guide conclusions. A classification and regression tree (CRT) was utilized for the decision tree analysis. To enhance the model’s robustness and ensure generalizability, cross-validation with a total number of ten sample folds was applied for model testing. This approach allowed for an unbiased evaluation of the tree's predictive performance and minimized overfitting.

## Results

### Cohort Overview

A total of 200,015 patients (n = 171,565 female, n = 28,148 male and n = 269 non-binary) with a mean age of 50.3 ± 14.5 years and mean BMI of 29.1 ± 6.8 kg/m^2^ were included in this analysis. The majority of patients were treated in an outpatient setting (n = 139,588; 69.8%) under general anesthesia (n = 191,323; 95.7%).

The mean preoperative laboratory values were: sodium 139.6 ± 2.7 mmol/L, BUN 14.7 ± 7.3 mg/dL, creatinine 0.8 ± 0.6 mg/dL, albumin 4.1 ± 0.6 g/dL, bilirubin 0.5 ± 0.4 mg/dL, SGOT 24.3 ± 20.4 U/L, alkaline phosphatase 80.3 ± 38.2 IU/L, WBC 6.9 ± 2.6 x10^9^/L, hematocrit 39.2 ± 4.4%, platelets 261.3 ± 75.1 x10^9^/L, PTT 29.7 ± 6.2 s, INR 1.0 ± 0.3 and prothrombin time 12.4 ± 2.8 s.

Complications were reported in 11.4% (n = 22,783) of cases, with surgical complications reported in 6.8% (n = 13,647) and medical complications in 2.1% (n = 4,122) of cases. Table 1 provides further details on postoperative complication occurrence.

**Table 1:** Summary of complications observed in the study

Any complication 11.4% (22,783)					
Mortality 0.2% (397)	Return to OR 4.4% (8,849)	Readmission 3.3% (6,607)	Unplanned readmission 3.0% (6,008)	**Surgical complications** 6.8% (13,647)	**Medical complications** 2.1% (4,122)
				Superficial surgical site infection 2.6% (5,123)	Pneumonia 0.3% (603)
				Deep surgical site infection 0.8% (1,616)	Reintubation 0.2% (316)
				Organ specific site infection 0.9% (1,782)	Pulmonary embolism 0.2% (381)
				Wound dehiscence 1.0% (2,079)	Failure to wean 0.2% (395)
				Blood transfusion 2.2% (4,330)	Renal insufficiency 0.1% (176)
					Dialysis 0.0% (100)
					Urinary infection 0.4% (853)
					CVA/Stroke 0.0% (79)
					Cardiac arrest requiring CRP 0.1% (127)
					Myocardial infection 0.1% (137)
					Deep vein thrombosis 0.3% (545)
					Sepsis 0.7% (1,439)
					Septic shock 0.1% (287)

Values are given as percentage (absolute number)

### Multivariate Binary Logistic Regression

The occurrence of any complication was found to be significantly influenced by the following laboratory values: elevated BUN [OR 1.019, 95% CI: 1.005–1.033, *p* = 0.009], reduced albumin [OR 0.460, 95% CI: 0.375–0.564, *p *< 0.001], elevated SGOT [OR 1.004, 95% CI: 1.000–1.008, *p *= 0.048], elevated WBC [OR 1.087, 95% CI: 1.046–1.130, *p *< 0.001], and reduced HCT [OR 0.915, 95% CI: 0.890–0.941, *p *< 0.001].

The results of the multivariate binary logistic regression analyses for each surgical and medical complications as well as for each specific complication are summarized in Table 2 and Supplementary Tables 1, 2, 3, 4, and 5. The absolute values for each risk group are summarized in Supplementary Table 6.

**Table 2:** Multivariate binary logistic regression for the occurrence of any, surgical and medical complications, for all preoperative laboratory values included in the analysis

	Any complication		Surgical complication		Medical complication	
	OR [95% CI]	*p value*	OR [95% CI]	*p value*	OR [95% CI]	*p value*
Sodium	1.007 [0.967–1.050]	0.724	0.981 [0.934–1.032]	0.460	1.042 [0.982–1.105]	0.175
BUN	1.019 [1.005–1.033]	**0.009**	1.016 [1.000–1.032]	0.056	1.027 [1.009–1.046]	**0.003**
Creatinine	0.922 [0.782–1.086]	0.331	0.992 [0.828–1.189]	0.931	0.812 [0.654–1.008]	0.059
Albumin	0.460 [0.375–0.564]	**< 0.001**	0.664 [0.517–0.853]	**0.001**	0.344 [0.255–0.464]	**< 0.001**
Bilirubin	0.985 [0.766–1.266]	0.905	1.045 [0.795–1.372]	0.754	1.168 [0.868–1.572]	0.304
SGOT	1.004 [1.000–1.008]	**0.048**	1.000 [0.996–1.004]	0.954	1.000 [0.994–1.006]	0.924
Alkaline phosphatase	1.001 [0.998–1.003]	0.568	1.000 [0.998–1.003]	0.822	1.001 [0.998–1.004]	0.393
WBC	1.087 [1.046–1.130]	**< 0.001**	1.037 [0.997–1.080]	0.071	1.086 [1.038–1.138]	**< 0.001**
HCT	0.915 [0.890–0.941]	**< 0.001**	0.933 [0.901–0.966]	**< 0.001**	0.933 [0.893–0.974]	**0.002**
Platelets	0.999 [0.998–1.001]	0.254	0.999 [0.998–1.001]	0.457	0.999 [0.998–1.001]	0.544
PTT	1.014 [0.998–1.030]	0.089	1.002 [0.983–1.022]	0.824	1.005 [0.984–1.026]	0.661
INR	1.749 [0.758–4.037]	0.190	1.150 [0.373–3.548]	0.807	2.209 [0.595–8.203]	0.236
Prothrombin time	0.970 [0.898–1.047]	0.431	0.996 [0.905–1.097]	0.940	0.945 [0.833–1.072]	0.380

Statistically significant values (*p *< 0.05) are indicated in bold

*OR* Odds ratio, *CI* Confidence interval

### Decision Trees

For the occurrence of any complication, the decision tree identified albumin levels as the most significant factor, forming the root node. A threshold of ≤ 3.3 g/dL split the cohort into two branches: patients with lower albumin levels who had a higher risk of complications (36.5%), and those with higher levels who showed a lower risk of complications (10.4%).

**Fig. 2:**
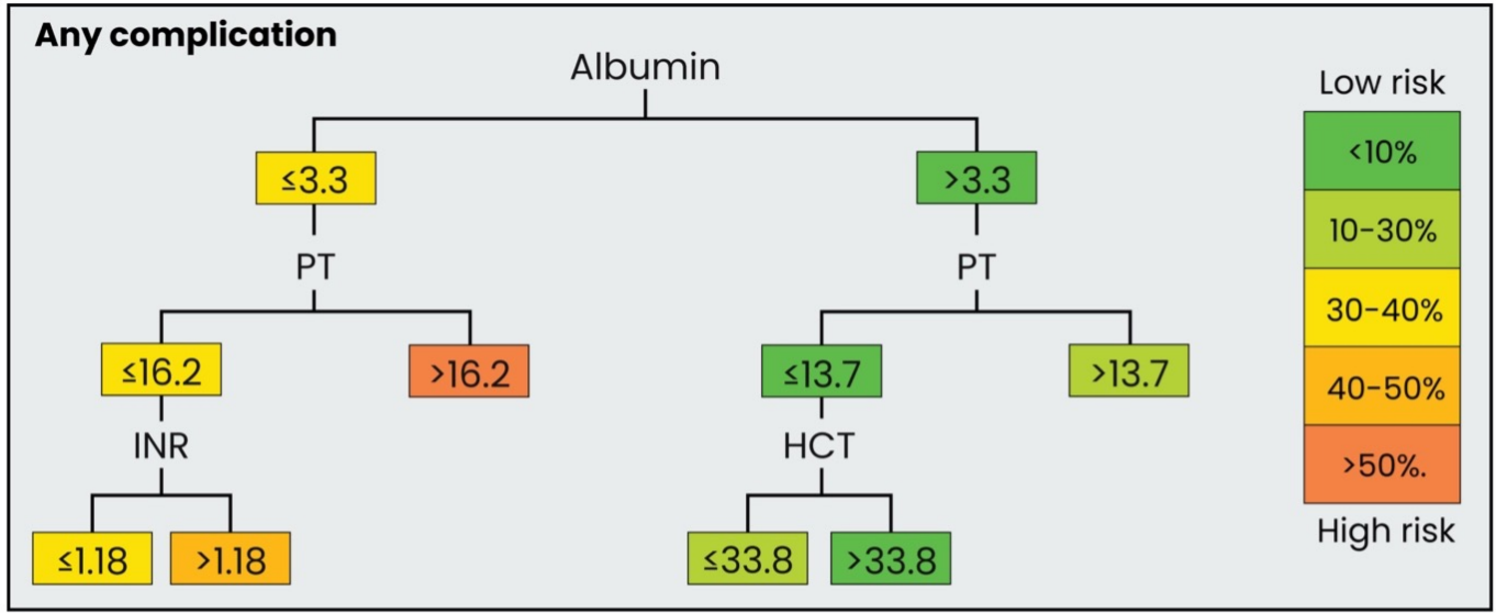
Simplified decision tree modeling of the likelihood for the occurrence of any complications using preoperative laboratory values

**Fig. 3:**
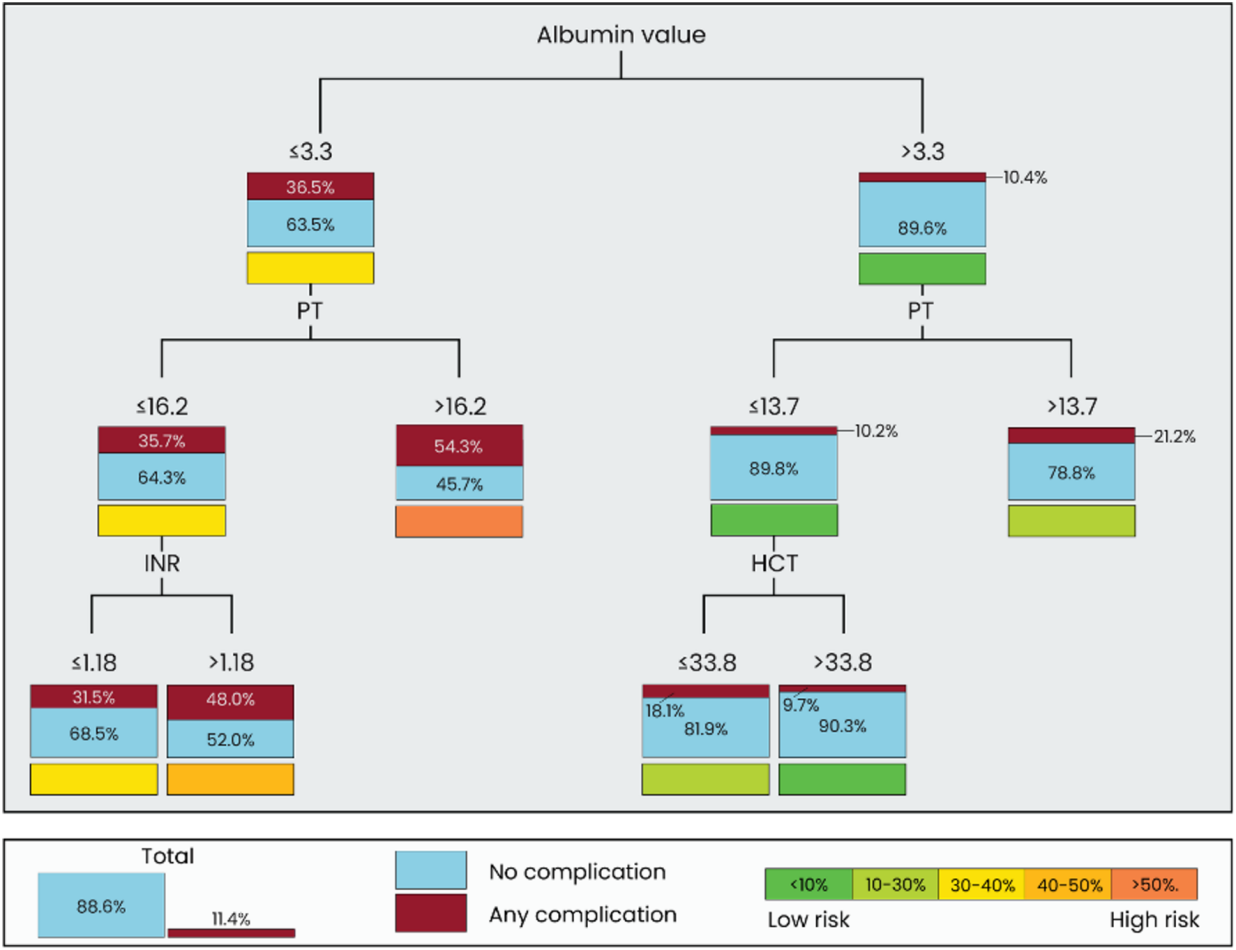
Detailed decision tree modeling the likelihood for the occurrence of any complication using preoperative laboratory values. Risk from low to high: < 10%, 10–30%. 30–40%, 40–50%, High risk: > 50%

For the high-risk group (albumin levels ≤ 3.3 g/dL), significantly higher complication rates were found for PT > 16.2 s (54.3%) compared to ≤ 16.2 s (35.7%). Patients with PT ≤ 16.2 s and an INR ≤ 1.18 were associated with lower rates of complications (31.5%), whereas INR > 1.18 indicated higher rates of complications (48.0%). For the low-risk group (albumin levels > 3.3 g/dL), PT remained a key predictor with ≤ 13.7 s indicating lower risk (10.2%) and >13.7 s indicating higher risk (21.2%). In patients with PT ≤ 13.7 s, HCT ≤ 33.8% slightly elevated risk compared to higher HCT values with 18.1 versus 9.7% complication rate (Figs. 2 and 3).

The decision trees summarizing the occurrence of surgical and medical complications is presented in Supplementary Figs. 1 and 2.

## Discussion

This multi-institutional analysis of 200,015 patients undergoing PRS procedures revealed that PLVs are valid predictors of complications. The employed database captures a wide array of surgeries ranging from aesthetic procedures such as breast augmentation, abdominoplasty and facial cosmetic surgery to complex reconstructive operations after trauma or oncologic resection including free tissue transfer as well as local and regional flap surgery. It further includes subspecialty domains such as hand surgery and burn reconstruction, extending across virtually every anatomical region. This diversity underscores the external validity of our findings: the identified laboratory predictors were consistent across a wide array of operative indications, surgical magnitudes, and patient populations and could therefore potentially pose as a benchmark for other surgical disciplines besides PRS. Albumin levels emerged as the most significant predictor across all decision trees. Specifically, patients with albumin levels ≤ 3.3 g/dL had a markedly higher risk of complications compared to those with higher levels. PT further stratified risk, with values >16 s in the high-risk group being associated with the highest complication rates.

In the multivariate analysis, reduced albumin, elevated BUN, elevated WBC, and reduced HCT consistently showed significant associations with adverse outcomes. The strongest predictors were hypoalbuminemia and elevated PT, highlighting the importance of these parameters in preoperative risk stratification and underscoring the need for targeted preoperative interventions to address out-of-range albumin, HCT, and coagulation parameters.

Comparable trends have also been reported in other surgical disciplines besides PRS, thereby supporting the generalizability of our findings. In gastrointestinal surgery, preoperative hypoalbuminemia predicts significantly higher rates of surgical-site infection and anastomotic complications. Further, the authors were able to demonstrate an inverse relationship between preoperative albumin levels and duration of inpatient stay, thereby effectively underlining the complex course in the operative and postoperative setting with increased morbidity [21]. The stark influence of hypoalbuminemia on wound infections was also reported in spine surgery where an albumin level of <3.5 g/dL was associated with a near 2.5-fold increase of surgical site infections. Another study by Chaker et al. even extended this threshold towards normal level and stated that borderline threshold of 3.5-4.0 g/dL were associated with increased complications [22, 23].

Albumin is a critical biomarker due to its various physiological functions. Albumin’s primary significance lies in its role as an indicator of nutritional status [24, 25]. Adequate nutrition is fundamental for wound healing, tissue regeneration, and collagen synthesis, all of which are pivotal in surgery—especially in PRS [26, 27]. Hypoalbuminemia is associated with impaired wound healing, which can compromise the success of procedures such as skin grafts, microsurgical flaps, or other reconstructive techniques that require precise tissue integration and wound healing [22, 28]. Beyond its nutritional implications, albumin also modulates the inflammatory response and supports various processes catering to the overall immune function. Hypoalbuminemia is often linked to a heightened inflammatory state, which can predispose patients to complications such as surgical site infections and delayed wound healing [29–32]. This inflammatory dysregulation is particularly relevant in PRS, where infections can lead to devastating outcomes, including flap necrosis, implant failure, or significant aesthetic compromise. Furthermore, albumin contributes to maintaining oncotic pressure within the vascular system, preventing excessive fluid leakage into surrounding tissues, which is of utmost relevance in reconstructive procedures where swelling can impair vascular perfusion and survival of microsurgical free flap procedures [33, 34]. Albumin also plays a key role in pharmacokinetics by binding to and transporting various drugs, including medication especially relevant for PRS inlcuding anesthetics and antibiotics. In hypoalbuminemic patients, altered drug binding and distribution can lead to inadequate therapeutic effects or increased drug toxicity, posing challenges during the perioperative period, where precise medication management is crucial to ensuring effective anesthesia, infection prevention, and pain control [35–37]. Conversely, pre-albumin has previously in the literature been discussed as an alternative marker of nutritional status due to its shorter half-life, higher sensitivity and greater responsiveness to short-term inflammatory and nutritional changes [38, 39]. While the authors fully acknowledge the potential value of this parameter, pre-albumin is not (yet) collected systematically within the ACS-NSQIP database and is typically measured only selectively, which could potentially introduce substantial selection bias. Albumin, on the other hand, is routinely available and standardized across the herein employed database, with the aim to reflect wider aspects of physiologic reserve relevant to surgical outcomes, such as nutritional status and systemic inflammation. Aligning with our objective to establish a pragmatic, easy-to-interpret and yet generalizable preoperative risk stratification tool based on *routinely* collected laboratory values, albumin was deemed to be the most appropriate marker.

In PRS, where optimal healing and minimal complications are critical, preoperative albumin optimization reduces perioperative risks and enhances surgical success [40–43]. Such preoperative strategies to raise albumin should first address reversible drivers such as the treatment of active infection and inflammation, optimization of hepatic function, and correction of malabsorption while reserving albumin administration for selected indications rather than as a stand-alone fix [44]. When preoperative preparation time allows, screening for malnutrition risk, for instance via the Malnutrition Universal Screening Tool (MUST), allows to initiate dietitian-led plans [45]. Effective treatments of infection and inflammation become crucial in the context since albumin as a negative acute phase protein inversely correlates with inflammatory state [46].

Beyond albumin, PT was identified as a key predictor of complications. For patients with low albumin levels, PT > 16 s was associated with significantly higher complication rates, particularly among those with PT values ≤ 23 s, who demonstrated the highest risk. Even with higher albumin, PT > 14 s indicated elevated risk. Prolonged PT, often reflecting coagulopathy or liver dysfunction, should be corrected to reduce perioperative bleeding. Preoperative options for PT correction include vitamin K supplementation or adjustment/temporary cessation of anticoagulant therapy. When time allows, these measures can be taken to allow for ideal conditions before elective surgery, while in urgent settings, prothrombin complex concentrates or plasma products may be used upon the surgeon’s and anesthesiologist’s discretion [47].

Lastly, HCT has also been identified to hold impact on preoperative risk stratification. In patients with albumin > 3.3 g/dL and PT < 14 s, otherwise classified as lower risk, HCT > 34% further delineated the subgroup with the lowest complication rates: A HCT of > 34% demonstrated the lowest risk. The importance of this parameter was corroborated in the multivariate logistic regression in which low HCT values were associated with a more likely occurrence of any, medical and surgical complications. Preoperative optimization strategies include a systematic hematologic diagnostic approach, incorporating parameters such as MCH, MCV, MCHC, and iron studies, thereby enabling a targeted treatment of underlying deficiencies. Embedding these measures within structured patient blood management programs further strengthens perioperative safety and reduces transfusion requirements [48, 49].

From a practical perspective, the thresholds identified in the decision tree provide actionable cutoffs for surgical planning. Specific cutoffs—as identified in the decision tree for the occurrence of any complication—for albumin (≤ 3.3 g/dL), PT (16 s), and HCT (34%) allow surgeons to stratify patients into higher-risk categories and determine the need for additional targeted optimization before surgery. Our findings call for a comprehensive preoperative workup that prioritizes albumin, PT, and HCT over more routine laboratory markers, which may not provide the same predictive value for complications. By systematically addressing these parameters, surgeons are enabled to reduce the likelihood of perioperative complications. Although these thresholds offer straightforward and readily interpretable guidance, their translation into clinical care must be seen with caution. It is important to note that these values should never be treated in isolation, as optimization must always address the patient in their entirety rather than an individual laboratory parameter. Finally, laboratory values in general represent only surrogate markers of complex physiological interplays. While they provide accessible and quantifiable thresholds, they capture merely the ‘tip of the iceberg’ and cannot fully reflect the multifactorial processes underlying the development of complications. Factors such as systemic inflammation, immune response, microvascular dynamics and wound biology remain only indirectly represented by these laboratory parameters. This underscores the need for cautious interpretation and future studies integrating more comprehensive biological and clinical data.

Despite the strength of these findings, optimizing out-of-range parameters remains largely underexplored in the literature, while albumin supplementation in surgical patients is debated due to conflicting evidence [44]. Hypoalbuminemia is not a standalone disease but rather a symptom of an underlying systemic condition. A 1998 meta-analysis, which reported increased mortality in critically ill patients receiving albumin supplementation, was one of the first to question the safety of this practice [50]. However, this analysis has since been challenged by numerous studies, including RCTs, that demonstrated benefits of albumin administration in patients with cirrhosis [51], sepsis [52], and burns [53]. More recently, however, a 2022 RCT found no reduction in Clavien–Dindo grade ≥ 2 complications with preemptive albumin in pancreatectomy patients [54]. Postoperative declines in serum albumin, driven by multiple factors such as fluid therapy, metabolic changes, surgery-inherent damage to the endothelial glycocalyx, and systemic stress, place patients with lower preoperative levels at higher risk. The role of perioperative albumin administration and strategies to enhance retention warrant further study. Anemia correction via iron supplementation, erythropoiesis-stimulating agents, or transfusions, on the other hand, is well established to improve surgical outcomes [55–58]. Likewise, for coagulation parameters, managing prolonged PT through targeted strategies such as Vitamin K administration or correcting underlying coagulopathies could further mitigate risk [59].

Although specific recommendations for preoperative optimization of these parameters are currently limited, recent guidelines, such as the Enhanced Recovery After Surgery [ERAS] protocol, emphasize the role of prehabilitation in improving surgical outcomes. These include preoperative nutritional screening and intervention for patients with hypoalbuminemia, as well as anemia management protocols [60, 61]. Further, implementing coagulation screening and correction protocols for at-risk patients should be considered a priority. However, these strategies need to be validated in future studies in PRS-specific populations to establish tailored guidelines.

## Limitations

This study is not without limitations. Firstly, the reliance on a single database, specifically the ACS-NSQIP, introduces potential bias, as it encompasses a select group of hospitals that may not represent the full range of clinical settings or patient populations. Additionally, the standardized nature of data collection within the database limits the availability of potentially relevant variables, such as a broader range of PLVs, detailed information on severity of comorbidities, surgical complexity, and comprehensive nutritional assessments. Moreover, variations in the quality, consistency, and frequency of data reporting across different institutions introduce additional complexities, which are impossible to fully account for in the analysis. These limitations constrain the depth of analysis and may exclude factors critical to risk prediction.

Secondly, variations in laboratory testing methodologies and reference ranges may influence both the measurement and interpretation of PLVs. This variability could affect the reproducibility and generalizability of the identified thresholds for risk stratification, limiting their applicability in universal institution-independent healthcare settings on a global scale.

Lastly, as a retrospective study, this analysis is inherently constrained by selection bias, reliance on pre-existing data, and the inability to establish causality between identified predictors and postoperative complications. Future multicenter, large-scale prospective studies are necessary to validate these findings, refine predictive thresholds, and establish causal relationships, thereby ensuring broader applicability.

## Conclusion

In summary, albumin is identified as an indispensable laboratory value in PRS due to its multifaceted roles in supporting nutrition, immune function, fluid balance, and drug pharmacokinetics, as well as its association with systemic health. Its consistent correlation with postoperative complications highlights its importance as a preoperative marker, emphasizing the need for targeted interventions to correct hypoalbuminemia and improve outcomes in this diverse and demanding surgical field. This analysis also underscores the critical role of albumin, PT, and HCT as key predictors of surgical and medical complications in PRS. Accordingly, preoperative optimization of these parameters—through nutritional support, coagulation management, and anemia correction—should be understood as a cornerstone of risk reduction strategies. By adopting an individualized, threshold-based approach, PRS surgeons can better manage high-risk patients, ensure safer surgical procedures, and achieve more favorable postoperative outcomes.

## Supplementary Information

Below is the link to the electronic supplementary material.

*Supplementary Table 1:* Multivariate binary logistic regression for the occurrence of mortality, reoperation, readmission and unplanned readmission, for all preoperative laboratory values included in the analysis. Statistically significant values (*p* < 0.05) are indicated in bold. OR, Odds ratio, CI, Confidence interval.

*Supplementary Table 2:* Multivariate binary logistic regression for the occurrence of specific surgical complications (superficial surgical site infection, deep surgical site infection, organ specific site infection, wound dehiscence, bleeding requiring transfusion), for all preoperative laboratory values included in the analysis. Statistically significant values (*p* < 0.05) are indicated in bold. OR, Odds ratio, CI, Confidence interval.

*Supplementary Table 3:* Multivariate binary logistic regression for the occurrence of specific medical complications (pneumonia, reintubation, pulmonary embolism, failure weaning, renal insufficiency), for all preoperative laboratory values included in the analysis. Statistically significant values (*p* < 0.05) are indicated in bold. OR, Odds ratio, CI, Confidence interval.

*Supplementary Table 4:* Multivariate binary logistic regression for the occurrence of specific medical complications (dialysis, urinary infection, CNS CVA, cardiac arrest, myocardial infarction), for all preoperative laboratory values included in the analysis. Statistically significant values (*p* < 0.05) are indicated in bold. OR, Odds ratio, CI, Confidence interval.

*Supplementary Table 5:* Multivariate binary logistic regression for the occurrence of specific medical complications (DVT, sepsis, septic shock), for all preoperative laboratory values included in the analysis. Statistically significant values (*p* < 0.05) are indicated in bold. OR, Odds ratio, CI, Confidence interval.

*Supplementary Table 6:* Absolute complication risks for the individual risk groups for any, surgical and medical complications.

*Supplementary Fig. 1* Detailed decision tree modeling the likelihood for the occurrence of surgical complications using preoperative laboratory values. Risk from low to high: < 10%, 10–30%. 30–40%, 40-50%, High risk: > 50%.

*Supplementary Fig. 2* Detailed decision tree modeling the likelihood for the occurrence of medical complications using preoperative laboratory values. Risk from low to high: < 10%, 10-30%. 30-40%, 40-50%, High risk: > 50%.
