# Supplementary material for: From Serum to Surgery: The Significance of Albumin in Preoperative Risk Stratification—An Analysis of 200,015 Plastic Surgery Patients
Source: Aesthetic Plast Surg. 2026 Mar 17;50(9):3530–40. doi: 10.1007/s00266-026-05800-8 (PMC13183695; doi:10.1007/s00266-026-05800-8)
Supplement: Supplementary file 1 — Supplementary Table 1: Multivariate binary logistic regression for the occurrence of mortality, reoperation, readmission and unplanned readmission, for all preoperative laboratory values included in the analysis. Statistically significant values (p < 0.05) are indicated in bold. OR, Odds ratio, CI, Confidence interval. [file 266_2026_5800_MOESM1_ESM.docx]

|  | Mortality | | Reoperation | | Readmission | | Unplanned Readmission | |
| --- | --- | --- | --- | --- | --- | --- | --- | --- |
|  | OR [95% CI] | *p value* | OR [95% CI] | *p value* | OR [95% CI] | *p value* | OR [95% CI] | *p value* |
| Sodium | 1.124 [1.018-1.242] | **0.021** | 0.956 [0.910-1.005] | 0.076 | 0.987 [0.858-1.137] | 0.859 | 0.954 [0.820-1.111] | 0.564 |
| BUN | 1.044 [1.019-1.069] | **<0.001** | 1.007 [0.991-1.023] | 0.397 | 1.026 [0.984-1.069] | 0.228 | 1.036 [0.992-1.082] | 0.107 |
| Creatinine | 0.787 [0.583-1.063] | 0.118 | 0.999 [0.832-1.201] | 0.995 | 0.752 [0.383-1.476] | 0.408 | 0.682 [0.330-1.411] | 0.303 |
| Albumin | 0.351 [0.218-0.568] | **<0.001** | 0.554 [0.432-0.709] | **<0.001** | 0.749 [0.378-1.487] | 0.409 | 0.723 [0.335-1.561] | 0.408 |
| Bilirubin | 1.156 [0.740-1.806] | 0.524 | 1.020 [0.777-1.337] | 0.889 | 0.643 [0.225-1.837] | 0.410 | 0.558 [0.160-1.948] | 0.361 |
| SGOT | 0.992 [0.985-0.999] | **0.036** | 1.003 [0.999-1.007] | 0.143 | 1.009 [1.003-1.015] | **0.002** | 1.009 [1.003-1.015] | **0.003** |
| Alkaline Phosphatase | 1.004 [1.001-1.007] | **0.021** | 0.999 [0.997-1.002] | 0.708 | 0.999 [0.991-1.007] | 0.804 | 0.998 [0.989-1.008] | 0.711 |
| WBC | 1.060 [0.999-1.123] | 0.052 | 1.022 [0.982-1.064] | 0.283 | 0.855 [0.718-1.017] | 0.076 | 0.904 [0.762-1.072] | 0.245 |
| HCT | 0.917 [0.854-0.985] | **0.017** | 0.934 [0.902-0.968] | **<0.001** | 0.868 [0.798-0.945] | **0.001** | 0.874 [0.798-0.958] | **0.004** |
| Platelets | 0.999 [0.996-1.002] | 0.384 | 1.000 [0.999-1.002] | 0.781 | 1.001 [0.997-1.006] | 0.555 | 1.000 [0.995-1.005] | 0.919 |
| PTT | 1.033 [1.004-1.063] | **0.025** | 1.014 [0.997-1.031] | 0.108 | 0.998 [0.941-1.058] | 0.939 | 1.002 [0.940-1.068] | 0.955 |
| INR | 3.192 [0.348-29.305] | 0.305 | 1.512 [0.559-4.094] | 0.416 | 1.678 [0.033-85.572] | 0.796 | 0.293 [0.002-53.094] | 0.643 |
| Prothrombin Time | 0.915 [0.729-1.147] | 0.439 | 1.016 [0.929-1.112] | 0.724 | 0.923 [0.657-1.296] | 0.642 | 0.934 [0.644-1.355] | 0.720 |
