# Supplementary material for: From Serum to Surgery: The Significance of Albumin in Preoperative Risk Stratification—An Analysis of 200,015 Plastic Surgery Patients
Source: Aesthetic Plast Surg. 2026 Mar 17;50(9):3530–40. doi: 10.1007/s00266-026-05800-8 (PMC13183695; doi:10.1007/s00266-026-05800-8)
Supplement: Supplementary file 2 — Supplementary Table 2: Multivariate binary logistic regression for the occurrence of specific surgical complications (superficial surgical site infection, deep surgical site infection, organ specific site infection, wound dehiscence, bleeding requiring transfusion), for all preoperative laboratory values included in the analysis. Statistically significant values (p < 0.05) are indicated in bold. OR, Odds ratio, CI, Confidence interval. [file 266_2026_5800_MOESM2_ESM.docx]

|  | Superficial  Surgical Site Infection | | Deep  Surgical Site Infection | | Organ Specific  Surgical Site Infection | | Wound Dehiscence | | Bleeding requiring transfusion | |
| --- | --- | --- | --- | --- | --- | --- | --- | --- | --- | --- |
|  | OR [95% CI] | *p value* | OR [95% CI] | *p value* | OR [95% CI] | *p value* | OR [95% CI] | *p value* | OR [95% CI] | *p value* |
| Sodium | 0.911 [0.820-1.011] | 0.080 | 0.882 [0.795-0.978] | **0.017** | 1.112 [0.917-1.348] | 0.281 | 1.085 [0.950-1.238] | 0.231 | 1.036 [0.965-1.113] | 0.326 |
| BUN | 1.002 [0.965-1.040] | 0.924 | 0.997 [0.964-1.031] | 0.844 | 1.047 [1.000-1.096] | **0.048** | 0.943 [0.876-1.014] | 0.112 | 1.024 [1.004-1.045] | **0.020** |
| Creatinine | 1.050 [0.695-1.585] | 0.818 | 1.292 [0.954-1.751] | 0.098 | 0.697 [0.337-1.442] | 0.330 | 0.989 [0.438-2.232] | 0.979 | 0.844 [0.646-1.102] | 0.212 |
| Albumin | 0.968 [0.564-1.662] | 0.906 | 0.486 [0.275-0.857] | **0.013** | 0.754 [0.293-1.937] | 0.557 | 0.653 [0.338-1.262] | 0.205 | 0.643 [0.460-0.898] | **0.009** |
| Bilirubin | 0.448 [0.138-1.454] | 0.181 | 0.992 [0.652-1.509] | 0.970 | 1.326 [0.624-2.817] | 0.464 | 0.348 [0.069-1.746] | 0.200 | 1.176 [0.843-1.642] | 0.340 |
| SGOT | 1.002 [0.993-1.012] | 0.661 | 1.002 [0.996-1.008] | 0.450 | 0.993 [0.965-1.023] | 0.658 | 0.987 [0.957-1.018] | 0.419 | 0.998 [0.992-1.004] | 0.476 |
| Alkaline Phosphatase | 1.000 [0.993-1.006] | 0.950 | 1.002 [0.998-1.006] | 0.268 | 1.002 [0.995-1.009] | 0.606 | 0.999 [0.988-1.009] | 0.807 | 1.000 [0.996-1.003] | 0.885 |
| WBC | 1.005 [0.915-1.103] | 0.923 | 1.008 [0.934-1.088] | 0.831 | 1.064 [0.947-1.194] | 0.297 | 1.088 [0.991-1.193] | 0.076 | 1.025 [0.973-1.078] | 0.353 |
| HCT | 0.988 [0.920-1.061] | 0.737 | 1.000 [0.921-1.085] | 0.996 | 0.959 [0.846-1.087] | 0.509 | 1.045 [0.950-1.150] | 0.363 | 0.851 [0.812-0.893] | **<0.001** |
| Platelets | 0.998 [0.994-1.002] | 0.303 | 0.997 [0.993-1.001] | 0.171 | 0.999 [0.992-1.005] | 0.646 | 1.001 [0.998-1.005] | 0.460 | 1.000 [0.998-1.002] | 0.727 |
| PTT | 1.000 [0.955-1.048] | 0.983 | 1.010 [0.974-1.047] | 0.591 | 0.971 [0.859-1.097] | 0.633 | 1.011 [0.961-1.063] | 0.672 | 1.002 [0.976-1.029] | 0.883 |
| INR | 3.414 [0.476-24.500] | 0.222 | 0.587 [0.035-9.703] | 0.710 | 0.987 [0.010-101.880] | 0.995 | 0.643 [0.015-27.782] | 0.818 | 0.842 [0.203-3.495] | 0.813 |
| ProthrombinTime | 0.893 [0.726-1.099] | 0.285 | 1.019 [0.816-1.271] | 0.870 | 0.978 [0.662-1.443] | 0.909 | 0.932 [0.706-1.230] | 0.618 | 1.070 [0.953-1.202] | 0.251 |
