# Supplementary material for: From Serum to Surgery: The Significance of Albumin in Preoperative Risk Stratification—An Analysis of 200,015 Plastic Surgery Patients
Source: Aesthetic Plast Surg. 2026 Mar 17;50(9):3530–40. doi: 10.1007/s00266-026-05800-8 (PMC13183695; doi:10.1007/s00266-026-05800-8)
Supplement: Supplementary file 4 — Supplementary Table 4: Multivariate binary logistic regression for the occurrence of specific medical complications (dialysis, urinary infection, CNS CVA, cardiac arrest, myocardial infarction), for all preoperative laboratory values included in the analysis. Statistically significant values (p < 0.05) are indicated in bold. OR, Odds ratio, CI, Confidence interval. [file 266_2026_5800_MOESM4_ESM.docx]

|  | Dialysis | | Urinary Infection | | CVA/Stroke | | Cardiac Arrest | | Myocardial Infarction | |
| --- | --- | --- | --- | --- | --- | --- | --- | --- | --- | --- |
|  | OR [95% CI] | *p value* | OR [95% CI] | *p value* | OR [95% CI] | *p value* | OR [95% CI] | *p value* | OR [95% CI] | *p value* |
| Sodium | 0.918 [0.726-1.161] | 0.475 | 1.056 [0.924-1.206] | 0.425 | 1.317 [0.693-2.501] | 0.401 | 1.218 [0.953-1.556] | 0.116 | 1.289 [0.888-1.871] | 0.182 |
| BUN | 0.996 [0.914-1.085] | 0.926 | 1.015 [0.969-1.063] | 0.532 | 1.078 [0.944-1.231] | 0.267 | 1.073 [1.027-1.122] | **0.002** | 0.992 [0.885-1.112] | 0.896 |
| Creatinine | 1.270 [0.589-2.739] | 0.542 | 0.542 [0.234-1.255] | 0.153 | 1.186 [0.300-4.684] | 0.807 | 0.490 [0.222-1.081] | 0.077 | 0.961 [0.296-3.120] | 0.948 |
| Albumin | 1.323 [0.348-5.029] | 0.681 | 0.391 [0.210-0.727] | **0.003** | 2.780 [0.094-81.984] | 0.554 | 0.289 [0.097-0.863] | **0.026** | 0.595 [0.139-2.546] | 0.484 |
| Bilirubin | 0.746 [0.182-3.054] | 0.684 | 1.222 [0.608-2.455] | 0.574 | 1.023 [0.082-12.701] | 0.986 | 0.685 [0.173-2.703] | 0.589 | 1.315 [0.189-9.143] | 0.782 |
| SGOT | 1.006 [0.994-1.019] | 0.306 | 0.994 [0.980-1.007] | 0.348 | 0.991 [0.954-1.029] | 0.639 | 0.996 [0.982-1.010] | 0.605 | 0.990 [0.964-1.018] | 0.492 |
| Alkaline Phosphatase | 0.995 [0.972-1.018] | 0.670 | 1.002 [0.997-1.008] | 0.441 | 0.999 [0.965-1.035] | 0.955 | 1.005 [1.000-1.011] | 0.055 | 1.003 [0.987-1.019] | 0.727 |
| WBC | 0.989 [0.867-1.128] | 0.872 | 1.041 [0.958-1.132] | 0.345 | 1.285 [0.975-1.695] | 0.075 | 0.986 [0.838-1.161] | 0.867 | 0.886 [0.606-1.295] | 0.531 |
| HCT | 0.789 [0.682-0.913] | **0.001** | 0.912 [0.831-1.002] | 0.056 | 1.160 [0.722-1.864] | 0.540 | 0.877 [0.739-1.041] | 0.133 | 0.887 [0.705-1.116] | 0.307 |
| Platelets | 0.998 [0.987-1.008] | 0.634 | 1.002 [0.999-1.005] | 0.166 | 1.000 [0.980-1.021] | 0.978 | 1.000 [0.994-1.006] | 0.918 | 1.000 [0.991-1.009] | 0.941 |
| PTT | 1.016 [0.946-1.091] | 0.660 | 1.031 [0.994-1.070] | 0.098 | 1.059 [0.842-1.331] | 0.625 | 1.036 [0.968-1.109] | 0.311 | 1.102 [1.045-1.162] | **<0.001** |
| INR | 8.912 [0.055-1442.740] | 0.399 | 0.471 [0.007-32.988] | 0.728 | 0.000 [0.000-2.756] | 0.081 | 6.829 [0.060-773.575] | 0.426 | 11.885 [0.072-1964.529] | 0.342 |
| ProthrombinTime | 0.983 [0.544-1.776] | 0.954 | 0.874 [0.630-1.213] | 0.419 | 1.099 [0.353-3.423] | 0.871 | 0.876 [0.520-1.477] | 0.620 | 0.838 [0.460-1.526] | 0.563 |
