# Supplementary material for: From Serum to Surgery: The Significance of Albumin in Preoperative Risk Stratification—An Analysis of 200,015 Plastic Surgery Patients
Source: Aesthetic Plast Surg. 2026 Mar 17;50(9):3530–40. doi: 10.1007/s00266-026-05800-8 (PMC13183695; doi:10.1007/s00266-026-05800-8)
Supplement: Supplementary file 5 — Supplementary Table 5: Multivariate binary logistic regression for the occurrence of specific medical complications (DVT, sepsis, septic shock), for all preoperative laboratory values included in the analysis. Statistically significant values (p < 0.05) are indicated in bold. OR, Odds ratio, CI, Confidence interval. [file 266_2026_5800_MOESM5_ESM.docx]

|  | DVT | | Sepsis | | Septic Shock | |
| --- | --- | --- | --- | --- | --- | --- |
|  | OR [95% CI] | *p value* | OR [95% CI] | *p value* | OR [95% CI] | *p value* |
| Sodium | 1.080 [0.948-1.230] | 0.246 | 0.961 [0.881-1.049] | 0.376 | 1.035 [0.920-1.165] | 0.567 |
| BUN | 1.014 [0.979-1.050] | 0.442 | 1.007 [0.979-1.035] | 0.634 | 1.023 [0.991-1.056] | 0.157 |
| Creatinine | 0.989 [0.642-1.524] | 0.962 | 0.815 [0.567-1.170] | 0.268 | 1.014 [0.721-1.424] | 0.938 |
| Albumin | 0.213 [0.110-0.410] | **<0.001** | 0.378 [0.245-0.583] | **<0.001** | 0.317 [0.169-0.596] | **<0.001** |
| Bilirubin | 1.328 [0.701-2.515] | 0.385 | 1.242 [0.791-1.948] | 0.347 | 1.396 [0.887-2.195] | 0.149 |
| SGOT | 0.996 [0.975-1.017] | 0.714 | 0.992 [0.976-1.008] | 0.317 | 0.994 [0.986-1.002] | 0.132 |
| Alkaline Phosphatase | 0.998 [0.991-1.005] | 0.572 | 1.001 [0.997-1.005] | 0.546 | 1.003 [0.998-1.007] | 0.229 |
| WBC | 1.031 [0.945-1.124] | 0.491 | 1.029 [0.971-1.091] | 0.334 | 1.067 [0.994-1.144] | 0.072 |
| HCT | 0.985 [0.899-1.079] | 0.744 | 0.898 [0.842-0.957] | **0.001** | 1.002 [0.909-1.105] | 0.964 |
| Platelets | 0.997 [0.992-1.001] | 0.124 | 1.000 [0.997-1.002] | 0.955 | 1.000 [0.996-1.004] | 0.977 |
| PTT | 0.920 [0.833-1.015] | 0.096 | 0.998 [0.962-1.036] | 0.917 | 1.032 [0.997-1.068] | 0.078 |
| INR | 0.785 [0.016-37.984] | 0.903 | 7.210 [1.034-50.300] | **0.046** | 6.330 [0.859-46.622] | 0.070 |
| ProthrombinTime | 0.918 [0.676-1.246] | 0.582 | 0.823 [0.675-1.003] | 0.054 | 0.779 [0.614-0.988] | **0.039** |
