# Supplementary material for: From Serum to Surgery: The Significance of Albumin in Preoperative Risk Stratification—An Analysis of 200,015 Plastic Surgery Patients
Source: Aesthetic Plast Surg. 2026 Mar 17;50(9):3530–40. doi: 10.1007/s00266-026-05800-8 (PMC13183695; doi:10.1007/s00266-026-05800-8)
Supplement: Supplementary file 6 — Supplementary Table 6: Absolute complication risks for the individual risk groups for any, surgical and medical complications. [file 266_2026_5800_MOESM6_ESM.docx]

| **Complication type** | **Lab values** | | | | **Absolute risk [in %]** |
| --- | --- | --- | --- | --- | --- |
|  | **Albumin [g/dL]** | **PT [s]** | **HCT [%]** | **INR** |  |
| **Any complication** | ≤3.3 |  |  |  | **36.5%** |
|  | >3.3 |  |  |  | **10.4%** |
|  | ≤3.3 | ≤16.2 |  |  | **35.7%** |
|  | ≤3.3 | >16.2 |  |  | **54.3%** |
|  | ≤3.3 | ≤16.2 |  | ≤1.18 | **31.5%** |
|  | ≤3.3 | ≤16.2 |  | >1.18 | **48.0%** |
|  | >3.3 | ≤13.7 |  |  | **10.2%** |
|  | >3.3 | >13.7 |  |  | **21.2%** |
|  | >3.3 | ≤13.7 | ≤33.8 |  | **18.1%** |
|  | >3.3 | ≤13.7 | >33.8 |  | **9.7%** |
| **Surgical complication** | ≤3.46 |  |  |  | **19.2%** |
|  | >3.46 |  |  |  | **6.1%** |
|  | ≤3.46 |  | ≤28.3 |  | **30.5%** |
|  | ≤3.46 |  | >28.3 |  | **14.9%** |
|  | ≤3.46 | ≤16.7 | ≤28.3 |  | **29.6%** |
|  | ≤3.46 | >16.7 | ≤28.3 |  | **46.2%** |
|  | >3.46 | ≤13.3 |  |  | **6.0%** |
|  | >3.46 | >13.3 |  |  | **10.0%** |
|  | >3.46 | ≤13.3 | ≤33.8 |  | **10.8%** |
|  | >3.46 | ≤13.3 | >33.8% |  | **5.7%** |
| **Medical complication** | ≤3.11 |  |  |  | **15.0%** |
|  | >3.11 |  |  |  | **1.6%** |
