# Supplementary figures and images for: From Serum to Surgery: The Significance of Albumin in Preoperative Risk Stratification—An Analysis of 200,015 Plastic Surgery Patients
Source: Aesthetic Plast Surg. 2026 Mar 17;50(9):3530–40. doi: 10.1007/s00266-026-05800-8 (PMC13183695; doi:10.1007/s00266-026-05800-8)

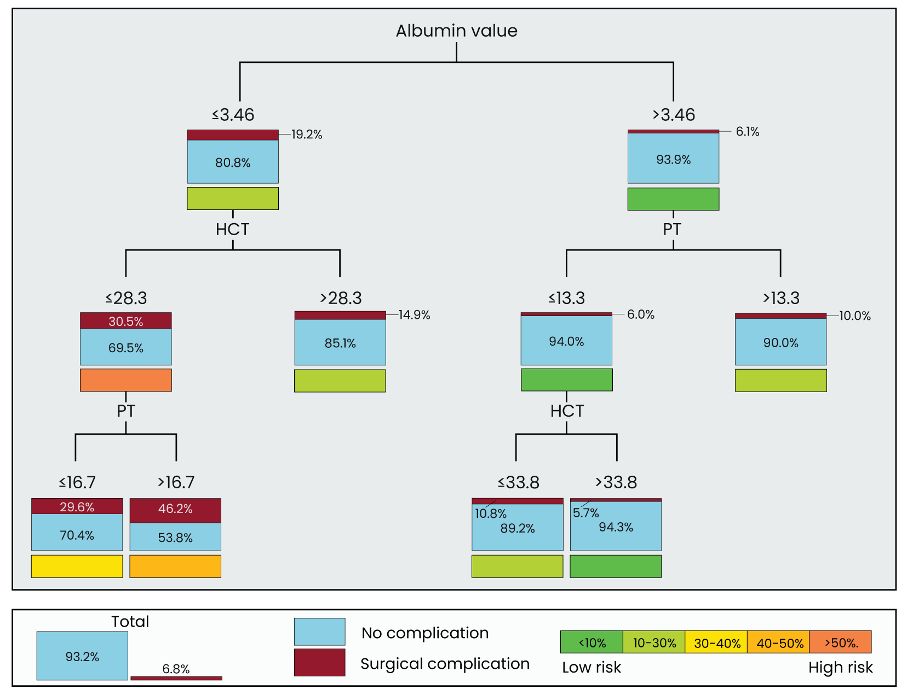

Supplement: Supplementary file 7 — Supplementary Fig. 1 Detailed decision tree modeling the likelihood for the occurrence of surgical complications using preoperative laboratory values. Risk from low to high: < 10%, 10–30%. 30–40%, 40-50%, High risk: > 50%. [file 266_2026_5800_MOESM7_ESM.png]

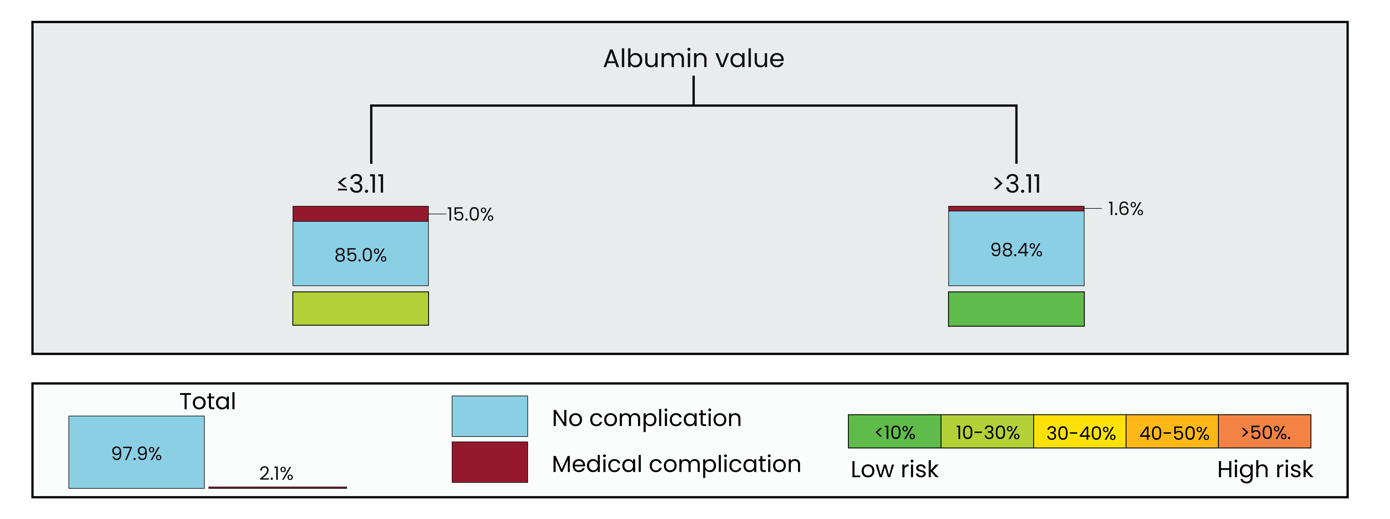

Supplement: Supplementary file 8 — Supplementary Fig. 2 Detailed decision tree modeling the likelihood for the occurrence of medical complications using preoperative laboratory values. Risk from low to high: < 10%, 10-30%. 30-40%, 40-50%, High risk: > 50%. [file 266_2026_5800_MOESM8_ESM.png]
